# A Review on Hemeoxygenase-2: Focus on Cellular Protection and Oxygen Response

**DOI:** 10.1155/2014/604981

**Published:** 2014-07-17

**Authors:** Jorge Muñoz-Sánchez, María Elena Chánez-Cárdenas

**Affiliations:** Laboratorio de Patología Vascular Cerebral, Instituto Nacional de Neurología y Neurocirugía Manuel Velasco Suárez, 14269 Delegación Tlalpan, DF, Mexico

## Abstract

Hemeoxygenase (HO) system is responsible for cellular heme degradation to biliverdin, iron, and carbon monoxide. Two isoforms have been reported to date. Homologous HO-1 and HO-2 are microsomal proteins with more than 45% residue identity, share a similar fold and catalyze the same reaction. However, important differences between isoforms also exist. HO-1 isoform has been extensively studied mainly by its ability to respond to cellular stresses such as hemin, nitric oxide donors, oxidative damage, hypoxia, hyperthermia, and heavy metals, between others. On the contrary, due to its apparently constitutive nature, HO-2 has been less studied. Nevertheless, its abundance in tissues such as testis, endothelial cells, and particularly in brain, has pointed the relevance of HO-2 function. HO-2 presents particular characteristics that made it a unique protein in the HO system. Since attractive results on HO-2 have been arisen in later years, we focused this review in the second isoform. We summarize information on gene description, protein structure, and catalytic activity of HO-2 and particular facts such as its cellular impact and activity regulation. Finally, we call attention on the role of HO-2 in oxygen sensing, discussing proposed hypothesis on heme binding motifs and redox/thiol switches that participate in oxygen sensing as well as evidences of HO-2 response to hypoxia.

## 1. Introduction

The hemeoxygenase (HO) system is composed by microsomal enzymes (EC 1.14.99.3; heme-hydrogen donor-oxygen oxidoreductases) involved in the degradation of heme, a molecule with important roles in biological systems. The HO system regulates hemeprotein levels and protects cells from the deleterious effects of intracellular free heme [1–3]. In mammalian cells, two catalytically active hemeoxygenase isozymes are expressed: HO-1 and HO-2.

HO-1 is a 32 kDa member of the stress protein superfamily (HSP32). It has a broad spectrum of inducers [4, 5] and is abundant in spleen, liver, and bone marrow [6]. On the other hand, the 36 kDa HO-2 does not respond to the numerous factors that induce HO-1. However, high expression of HO-2 is observed in testis, brain, and endothelial and smooth cells from cerebral vessels [7–10]. HO-1 has been the focus of researchers for a long time. Several studies report that overexpression of HO-1 mediated by various stimuli provides antioxidant protection in a variety of cells and tissues [6]. Due to its ability to respond to several cellular stresses and the numerous evidences of cell and tissue protection as result of HO-1 induction in different models and pathologies it has been considered as an ideal cytoprotective enzyme. Actually, it has been suggested that modulation of HO-1 expression and activity could have a potential therapeutic value [11–15]. In contrast, HO-2 due to its apparently constitutive role has been less studied. HO-2 has attracted the attention of several research groups since evidence obtained with genetically modified animals has revealed its protective relevance [16–18]. Furthermore, it has been observed that the expression of HO-2 in* in vivo* and* in vitro* models is not strictly constitutive as always stated [19–21]. Emblematic reviews have been published about the HO system [1, 7, 8, 22–24] and numerous reviews exist on HO-1 suggesting its protective role ([11–15, 25–28], between many others). However, our perception is that specific information about HO-2 has been less attended or at least only included as part of the HO system. We consider that there is enough and relevant information on HO-2 specifically. In this review, after a brief description of HO system as the main detoxifying system of heme to establish its cellular and biological importance, we focused in literature about HO-2 gene, protein structure, and catalytic activity. Finally we summarize evidence about suggested functions of HO-2 such as its protective role in cellular damage and importantly in the role of HO-2 as an enzyme that participates in the regulation of O_2_ sensing system and its response in models that generate hypoxic stress.

## 2. Heme and Its Degradation by the HO System

Heme is an iron-protoporphyrin complex with essential roles in biological systems. It is an essential prosthetic group of enzymes with functions such as oxygen storage and transport (hemoglobin and myoglobin), electron transport and energy generation (NADPH oxidase, guanylyl cyclase and cytochrome P450 family); and enzymatic systems such as catalase, peroxidase, nitric oxide synthase (NOS), and cyclooxygenase [3]. Several pathological conditions have shown the damaging effects of free heme [29–31]. The excess of free heme provokes cell damage and tissue injury particularly when is released from intracellular hemeproteins. The extreme hydrophobicity of heme may promote deleterious iron-dependent reactions leading to reactive oxygen species (ROS) generation and membrane lipid peroxidation, disrupting cellular membranes of several organelles such as mitochondria, endoplasmic reticulum, nuclei, and cell membrane [32, 33]. The cellular free heme may increase after extracellular heme overload, increased heme synthesis, accelerated breakdown of heme proteins by a cellular stress, or impaired incorporation into apo-heme proteins resulting in increased levels of ROS and subsequently, oxidative damage and cellular injury [3, 31]. Therefore, the levels of heme are tightly maintained and regulated by either synthesis or degradation mechanisms. In order to survive heme toxicity, cells have developed heme detoxification systems. In mammals, the main responsible to detoxify free heme is HO system, however Xanthine Oxidase and H_2_O_2_ plus NADPH-cytochrome P450 reductase can also degrade the heme molecule [8]. There are also extra heme degrading systems such as hemopexin, the formation of heme and albumin complexes to prevent the presence of extracellular heme in blood plasma, the heme-scavenger function of Lipocalin alpha 1-microglobulin (alpha-1 m), reduced glutathione able to degrade heme, and heme binding proteins [3].

**Figure 1:**
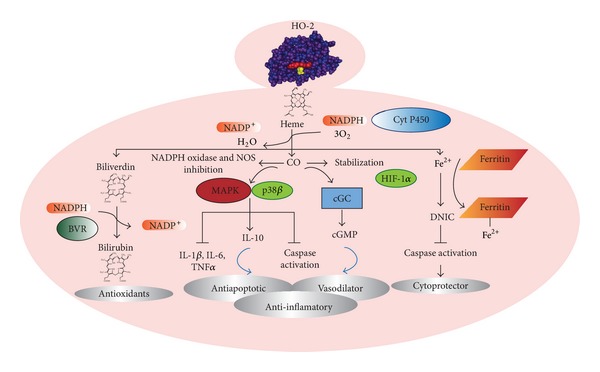
Physiological roles of HO-2 enzymatic activity products. Heme is cleaved by HO-2 producing equimolar amounts of CO, Fe^2+^, and BV. Enzymatic reaction requires NADPH, Cyt P450, and O_2_. CO has antiapoptotic, antiproliferative, and anti-inflammatory activities through p38/MAPK processes. CO also regulates vascular tone via cGC/cGMP, stabilization of HIF-1*α* and inhibits NADPH oxidase and NOS. BV is metabolized to BR by NAD(P)H : BVR. Fe^2+^ is sequestered by ferritin but also can form DNIC, a complex with protective effects through inhibition of caspases activity. BVR: biliverdin reductase; cGC: guanylate cyclase; DNIC: iron-sulfur cluster dinitrosyl iron-sulfur complex; HIF-1*α*: alfa subunit of Hypoxia inducible factor-1; NO: nitric oxide; and NOS; nitric oxide synthase.

The cytoprotective role of HO system comprises the reduction of cellular free heme and the generation of metabolites during the catalytic reaction. HO isoenzymes are positioned within endoplasmic reticulum, and in conjunction with NADPH cytochrome P450 reductase, split the tetrapyrrole heme ring to biliverdin (BV), free ferrous iron (Fe^2+^), and carbon monoxide (CO). BV is subsequently metabolized to bilirubin (BR) by Biliverdin Reductase (BVR) (Figure 1) [34].

**Figure 2:**
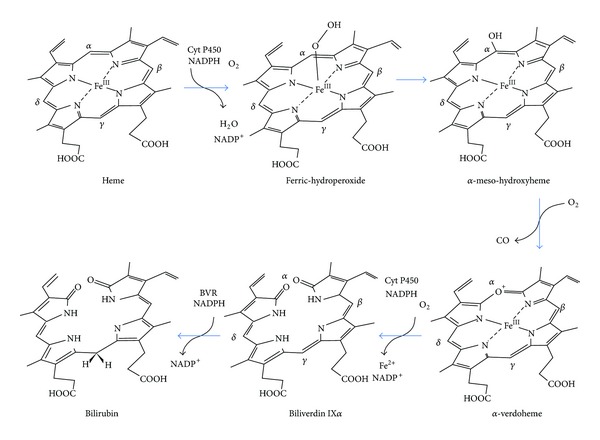
Catalytic reaction of HOs and BVR. The sequence is shown in three stages, separated by three well-defined intermediates: ferric-hydroperoxide, *α*-meso-hydroxyheme, and *α*-verdoheme. The final products of the reaction are CO, biliverdin IX*α*, and Fe^2+^. Biliverdin IX*α* is then reduced by BVR.

Reaction starts with the formation of the ferric heme-HO complex. Ferric heme-iron is then reduced to a ferrous state by the first electron donated from NADPH by NADPH cytochrome P450 reductase. Molecular oxygen binds to the complex to form a metastable oxy-form and iron-bound oxygen is converted to a hydroperoxide intermediate (Fe^3+^-OOH), by receiving another electron from the NADPH cytochrome P450 reductase and a proton from the distal pocket water. Terminal oxygen of Fe^3+^-OOH attacks the *α*-meso-carbon of the porphyrin ring to form ferric *α*-meso-hydroxyheme. This species of heme reacts with molecular oxygen and yields the ferrous verdoheme-HO complex and CO. The oxy-verdoheme-HO complex is converted to ferric iron-biliverdin chelate via a hydroperoxide intermediate, which is still bound to HO protein. The iron of the ferric biliverdin is reduced to the ferrous state by the reductase in order to liberate Fe^2+^ and BV to complete the total HO reaction (Figure 2). In the* in vitro* reaction, the release of BV from the enzyme is the rate limiting step [35–37].

### 2.1. The Relevance of HO Catalytic Activity Products

All the products of HO activity are involved in physiological and pathophysiological processes including fundamental adaptive response to cellular stress, apoptosis, and inflammation [38–41] (Figure 1). The bile pigments BV and BR are powerful antioxidants which scavenge ROS and nitrogen reactive species through recycling mechanism between BR and BV [42]. In addition, BR inhibits the activity of NADPH oxidase [43] and the complement cascade at the C1 step in the Classical Pathway, suggesting that bile pigments could serve as endogenous tissue protectors through anticomplement actions [44]. It has been reported that BR suppresses the inflammatory response inhibiting IL-1 and IL-2 production, decreasing natural killer activity and antibody dependent cellular toxicity [45].

Several physiological roles for CO, the other product of HO reaction, have been reported. One of them directly involve the modulation of guanylate cyclase with the resultant production of cyclic guanine monophosphate (cGMP) leading to vasodilation, neurotransmission, inhibition of platelet aggregation, and antiproliferative effects on smooth muscle [46]. CO also has shown anti-inflamatory, antiapoptotic, and antiproliferative effects through modulation of p38*β* and mitogen-activated protein kinase (MAPK) pathways [47–50]. A recent described role of CO is the cell protection by the stabilization of the hypoxia induced factor 1*α* (HIF-1*α*) in macrophages as well as in tissues from ischemia-reperfusion injury [51]. Additionally, CO protective effects were observed in endothelial cells, macrophages, and vascular smooth muscle cells attributed to a decrease in ROS generation* via* inhibition of cytochromes on both NADPH oxidase and the respiratory chain [52]. Higher levels of CO also inhibit NOS reducing the production of peroxynitrite [46].

Finally, Fe^2+^ produced by HO activity is rapidly removed by ferritin, limiting its pro-oxidant capacity [53]. Fe^2+^ released from heme metabolism interacts with intracellular thiols and forms iron-sulfur. The nitrosylated form of the iron-sulfur cluster dinitrosyl iron-sulfur complex (DNIC) endogenously protects cells from NO-induced toxicity by either scavenging NO or converting NO to a potent S-nitrosylating species. The participation of DNIC on different protective pathways has been suggested since the S-nitrosylating species can inhibit activity of caspases through the S-nitrosylation of their active site Cys residue [54, 55] (Figure 1).

### 2.2. Elements of the HO System

The isoform 1 of the HO system was characterized as an enzyme entity in 1974 [56, 57]. Later, the characterization of two HO independent forms was reported [58]. The authors purified and characterized HO isoforms from liver microsomes and reported the differential response of both enzymes to different purification steps, thermolability, activity and response to cobalt, cadmium, hematin, phenylhydrazine, and bromobenzene. This was also the first time that the two isoenzymes were proposed as different entities and named as HO-1 and HO-2. The same group reported the complete purification and characterization of HO-2 isoform from rat testis [59]. A third isoform was described [60]; however it has been characterized in rats as a pseudogene derived from HO-2 transcripts [61].

To date, it is known that HO isoenzymes are microsomal proteins that catalyze the same reaction. They are the product of independent genes and there is no resemblance between the two isoforms at gene structure, regulation, or tissue expression patterns [8, 23]. In fact, it has been suggested that HO isoforms might have different cellular functions and physiological roles [24, 62].

HO-1 has a broad spectrum of inducers, including heme (its natural substrate), transition metals, H_2_O_2_, *β*-amyloid, dopamine, kainic acid, cytokines, prostaglandins, endotoxin, and vasoactive compounds as well as other pro-oxidants and inflammatory stimuli. It is also induced by heat shock, radiation, hypoxia and hyperoxia [4, 5, 8]. HO-1 is abundantly expressed in spleen and other tissues that degrade senescent red blood cells, including specialized reticuloendothelial cells of the liver and bone marrow [6]. In fact, spleen is the only organ in which HO-1 is detected in unstressed conditions [8].

On the other hand, HO-2 has been considered as the constitutive isoform. High expression is observed in testis, brain, and endothelial and smooth cells from cerebral vessels [9, 10]. HO-2 is the abundant isoform in rat adult brain. It has been detected in neuronal populations in forebrain, hippocampus, midbrain, basal ganglia, thalamic regions, cerebellum, and brain stem (reviewed in [8]). In contrast, HO-1 is barely detected in brain tissue in the absence of stress inducers.

In the next section we recapitulate known information about the HO-2 isoform. HO-1 descriptions and comparisons when describing HO-2 are inevitable. However, next sections do not pretend to be a work on the vast information on HO-1, but a specific description of the existing information of HO-2 gene, transcript, as well as protein structure and regulation of HO-2.

## 3. Hemeoxygenase 2

### 3.1. HO-2 Gene

HO-1 and HO-2 are single copy genes that differ in gene structure and organization. HO-1 is encoded on chromosome 22 (22q12) in the human genome [71]. It is approximately 14 kb long, contains 5 exons, results from a single message of 1.8 kb, and translates to a 32 kDa protein [72]. HO-2 is encoded on chromosome 16 (16q12) [71] and has a complex organization. McCoubrek and Maines reported in 1994 the characterization and structure organization of the rat* Hmox2*. They showed that* Hmox2* has a length of 12,563 pb, possesses 5 exon and 4 intron regions, and lacks a conventional TATA box.

**Figure 3:**
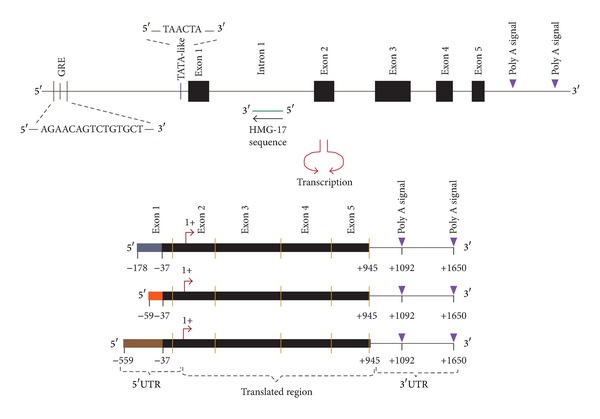
Structure of the* Hmox2* and multiple transcripts of rat HO-2. Introns are denoted by thin lines, coding sequences by black boxes. In the promoter sequence, the TATA-like sequence is observed. The upstream regulatory sequence GRE is shown. Nested sequence HMG-17 cDNA is indicated by a green line. Two potential polyadenylation signals are located in the 3′UTR in a purple arrow. The start codon is denoted as +1. Three different transcripts are identified as result of sequence variability in the upstream region from nucleotide −37. Figure based on information reported by [63–65].

Coding sequence of* Hmox1 *begins in exon 1, while the* Hmox2 *start codon is found in exon 2.* Hmox2 *exon 1 is composed entirely of the noncoding sequence 5′UTR [63] (Figure 3).

Particularly,* Hmox2* presents a nested sequence of 1046 nucleotides in the intron 1 with 87% identity to the cDNA encoding the mouse and human nonhistone chromosomal protein, HMG17 (Figure 3); a protein that facilitates the catenation of double-stranded DNA catalyzed by various topoisomerases and may confer specific conformations to transcribed regions in the genome [63].

### 3.2. HO-2 Transcripts and Regulation

In contrast to HO-1, which results from a single message [57], HO-2 is the product of two or more transcripts that translates a uniquely protein of 36 kDa. In most tissues, cell types, and mammalian species including human, two different sizes of 1.3 and 1.7–1.9 kb HO-2 transcripts have been identified. However, in rat, the presence of five HO-2 different transcripts with developmental and tissue-specific regulation has been demonstrated. The 1.45, 1.7 and 2.1 kb transcripts are unique to testis and expressed only in adulthood; the 1.45 transcript is the predominant form in testis, while 1.3 and 1.9 kb are common to every tissue examined [63, 64, 73]. In human, there are two HO-2 transcripts (1.3 and 1.7 kb) [65], while mice show two mRNAs named HO-2a and HO-2b [74]. Only one transcript of 1.7 kb has been identified in the monkey* Cebus apella* [75].

The different HO-2 transcripts result from processes such as differential initiation of transcription, alternative use of polyadenylation (poly A) sites or differential splicing, and stage-specific exon utilization. The identification of three different 5′UTRs sequences in the untranslated first exon of the rat transcripts indicates alternative splicing in* Hmox2 *mRNA processing. This, plus the presence of two poly A signals separated by 560 nucleotides on* Hmox2*, allows generation of transcripts of different sizes [64] (Figure 3). The presence of secondary stem/loop structures between the two HO-2 poly A signals suggests that these loops provide binding sites for proteins that could be controlling RNA stability and translation.

Differential use of the poly A signals was determined in several tissues of rat. Relative abundance of the 1.3 and 1.9 kb transcripts varied in a tissue-specific manner, with approximately equal amounts of both messages in kidney and brain. In testis, the smaller transcript predominates, while the larger transcript is scarcely detected. In liver, only the smaller transcript was detected. Additionally, the translation analysis showed that the 1.3 kb transcript is more efficiently translated than the 1.9 kb transcript [73], suggesting posttranscriptional regulatory mechanisms through poly A signals [64, 65].

One of the most important differences between HO-1 and HO-2 arises from the presence of different regulatory elements in the promoter region. Control of HO-1 transcription is complex and tightly regulated, with differences in expression found between tissues and species. As mentioned before, a wide variety of stimuli induce HO-1 expression. Multiple regulatory elements control human HO-1 gene transcription. These elements contain numerous transcription factor consensus binding sites in both the proximal and distal 5′ promoter sequences, as well as in an internal enhancer region. HO-1 gene expression can be upregulated through its multiple stress response as well as by numerous important transcription factors including Jun B, activator proteins 1 and 2 (AP-1 and 2), NF-*κ*B, HIF-1, and Nrf2 [6, 76, 77]. Conversely, the transcription factors Bach1 and Jun D act as negative regulators of human HO-1 gene expression [78, 79]. In contrast to these various regulatory elements in* Hmox1 *promoter, the only demonstrated functional response element in* Hmox2 *promoter is the glucocorticoid response element (GRE) [80]. Enhanced transcription by GRE regulation has been demonstrated with corticosterone or dexamethasone treatment in brain of old or newborn rat, respectively, where an increase of HO-2 transcripts (1.3 and 1.9 kb) and HO protein levels was observed [80, 81]. A remarkable increase of the tissue specific 2.1-and-1.45 kb transcripts was observed in newborn rat testis [82] after corticosterone administration, suggesting the direct involvement of the adrenal glucocorticoids in* Hmox2 *modulation.

### 3.3. HO-2 Protein Structure

HO-1 and HO-2 isoenzymes are homologous proteins that align in sequence and share the same fold and activity. The sequence identity of both proteins is 45% between full-length HO-1 and HO-2 and 55% in the conserved core regions. The core domains of both enzymes share a similar fold [66]. Crystal structure of the heme-free and-bound crystal structures of human HO-1 were described since 1999 [67]. More recently, Bianchetti et al. (2007) reported the apo- and heme-bound crystal structure of human HO-2 in a truncated form [66]. Additionally, the crystal structure of HO-2 from* Synechocystis sp.* PCC 6803 has been reported [83].

**Figure 4:**
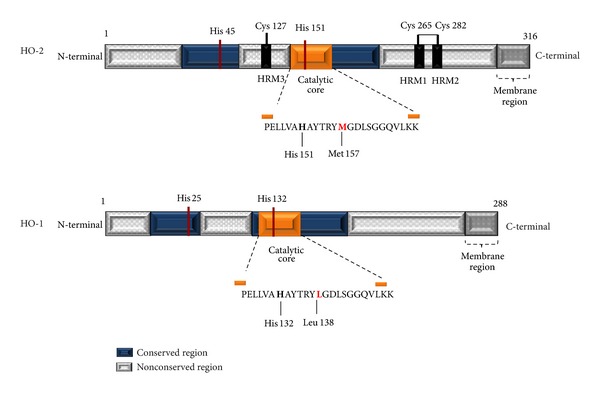
Comparison between HO-1 and HO-2 primary structures. Figure based on information reported by [66–68].

Crystallographic studies of HOs revealed that proteins are mostly *α*-helical with short loop segments that connect adjoining helices. In general, the secondary structure of HO-2 closely resembles apo- and heme-bound HO-1 [67, 83]. Primary sequence alignment shows that HO-1 and HO-2 exhibit two regions of sequence divergence: one is around residue 127 and the other is near the C-terminal between 240 and 295 (HO-2 numbering) (Figure 4) [66, 69]. Evidences show that differences in C-terminal of HO-1 and HO-2 play regulatory roles. It has been reported that HO-1 undergoes a regulated intramembranal proteolysis of 52 amino acids at C-terminal in order to be translocated to the nucleus and it has been hypothesized that HO-1 alters binding of transcription factors that respond to hypoxic or oxidative stress conditions affecting gene expression [84]. This proteolysis and nuclear translocation occurs in hypoxic conditions and is a specific behavior of HO-1 [85]. On the other hand, HO-2 in its C-terminal region contains particular sequences between residues 255 and 287 named the heme regulatory motifs (HRMs) (see below).

The catalytic core of HO-1 and HO-2 includes a 24 amino acid segment in the primary structure (that corresponds to residues 146 to 169 in human HO-2 and 126 to 149 in HO-1) perfectly conserved among all forms [8]. The only exception is the conservative substitution of a Leu residue found in all the known HO-1 proteins for a Met residue in HO-2. This segment is hydrophobic and forms a heme pocket that binds the heme pyrrole rings 1 and 2 (with vinyl side chains) through electrostatic interactions. The conserved His 132 in HO-1 facilitates heme catalysis while in HO-2 (His 151) is essential for catalytic activity [86, 87] (Figure 4). The heme pocket is formed by two helices named proximal and distal. The proximal helix provides the residue that stabilizes heme into catalytic core (His 25 for HO-1 and His 45 for HO-2) coordinating heme iron through the *τ* nitrogen of imidazole [66, 88]. Additionally, the carboxyl oxygen from Glu 49 forms a hydrogen bond with the *π* nitrogen of imidazole from His 45 in order to stabilize the iron-coordinating His. Gly 163 moves away from the heme, causing the heme pocket to adopt the open conformation, which is proposed to be important in binding heme and/or releasing products [66].

Although the C-terminal HRM region shows the most significant sequence difference between HO-1 and HO-2, most of the key amino acids involved in heme binding, Lys 38, His 45, Glu 49, Tyr 154, Gly 159, Asp 160, Gly 163, Lys 199, and Arg 203 are observed in similar positions in the heme-bound HO-2 structure when compared with heme-bound HO-1. The major differences in the crystal structure of apo-HO-2 and heme-bound HO-1 are seen in the helices that directly interact with the heme. The proximal helix of apo-HO-2 moves slightly downward and the distal helix moves upward relative to heme-bound HO-1. In heme-bound HO-2, the same displacement of the distal helix is observed when compared with heme-bound HO-1 [66].

#### 3.3.1. The Heme Regulatory Motifs in HO-2

A distinctive feature of HO-2 despite the high sequence identity and three-dimensional homology in the core domains between HO-1 and HO-2 is the presence of the HRMs in HO-2 described in 1997 [89]. These motifs are known to control processes related to iron and oxidative metabolism in organisms ranging from bacteria to humans. A HRM consists of a conserved Cys-Pro dipeptide core sequence that is usually flanked at the N-terminal by basic amino acids and at the C-terminal by a hydrophobic residue. HRM/heme interactions have been proposed to regulate the activity and/or stability of proteins involved in respiration and oxidative damage, coordination of protein synthesis and heme availability in reticulocytes, and iron and heme homeostasis [90–92]. To date, only eight proteins that possess HRM domains have been identified. These proteins have a heme/oxygen regulatory function in the cell such as aminolevulinate synthase-1 and 2, Bach-1 transcriptional regulator of HO-1 expression, eukaryotic Initiation factor 2 (eIF2) Kinase, transcriptional activator Hap1, Iron regulatory protein 2 (IRP-2), iron responsive regulator (IRR), periodic-2 circadian regulator (Per2), and HO-2 [68, 89].

HO-2 contains two conserved HRMs involving Cys 265 in HRM1 and Cys 282 in HRM2. A third HRM (Cys 127) has also been reported for HO-2, however its function is unknown [69]. The group of Maines described that these two HRMs bind heme with high affinity but are not involved in heme catalysis [89, 93]. However, it was shown recently that the HRMs in HO-2 do not bind heme* per se* but form a reversible thiol/disulfide redox switch that indirectly regulates HO-2 activity modulating substrate affinity. When the Cys of HRMs are in the reduced dithiol state, *K*_*d*_ value for the HO-2-heme complex is around 350 nM. This value drops significantly to 33 nM, similar to the intracellular free heme level, when the HRMs switch to the oxidized disulfide state, indicating a much stronger affinity of oxidized HO-2 for its substrate. Under oxidative stress conditions, HO-2 can degrade heme rapidly, increasing the levels of CO and BV; however, hypoxic or reduced conditions increase cellular free heme [68, 89, 94]. Recent results of Varfaj et al. have suggested that HRM heme affinity is not physiologically relevant [95]; however, the hypothesis of involvement of HRMs in redox switch and HO-2 participation in sensor oxygen system through BK_Ca_ channel activity (see below) is supported by numerous evidences.

#### 3.3.2. Modulation of HO-2 Activity

Abundant reports about the increase in HO-1 activity through enzyme induction exist. In contrast, there is a lack of information about HO-2 activation, with only a few reports appearing in the literature, which suggest that HO-2 activity can be modified by different mechanisms.

**Figure 5:**
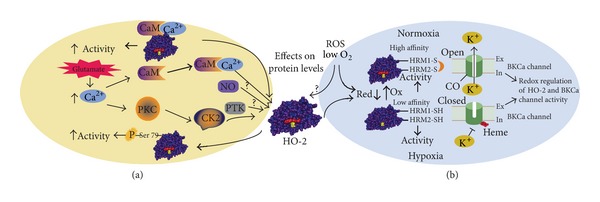
Schematic representation of (a) enzimatic activity regulation; (b) redox regulation of HO-2 and BK_Ca_ channel. (a) Posttranslational modifications of HO-2 such as Ser 79 phosphorylation via PKC/CK2 can be activated by Glutamate/Ca^2+^, increasing HO-2 enzimatic activity. CaM/Ca^2+^ complex also can increase HO-2 activity through interaction between CaM and HO-2 due to an increase of Ca^2+^ by Glutamate. PTK may increase the activity of HO-2. (b) HO-2 and BK_Ca_ constitute a universal oxygen sensor system. Normoxic conditions: BK_Ca_ channel opens because inhibitor heme has dissociated from the channel or because CO generated by HO-2 is bound. In hypoxic conditions, CO levels are low and heme is bound to channel, hence BK_Ca_ is closed. Thus, heme is bound to HO-2 under normoxic conditions and to the HBD of the BK_Ca_ channel under hypoxia. Based on hypothesis proposed by Yi and Ragsdale 2007 [69] Williams et al. 2004 [70]; see text for details. BK_Ca_ channel: voltage- and Ca^2+^-activated large conductance K^+^ channel; CaM: calmodulin; CK2: Casein Kinase 2; PKC: Protein Kinase C; and PTK: Protein Tyrosine Kinase.

It has been established that HO-2 activity is substrate dependent and that factors that increase heme substrate availability also increase CO production [96]. HO-2 activity can be affected by posttranslational modifications.* In vitro* studies have shown that HO-2 activity is influenced by CK2-dependent phosphorylation at Ser 79 and that PKC protein could be implicated in this mechanism. In hippocampal culture, activation of PKC* via* phorbol ester treatment directly phosphorylates and activates CK-2, which in turn phosphorylates and activates HO-2 [97]. Authors suggested that HO-2 activation* via* PKC during neuronal depolarization could also involve Ca^2+^ entry. Later they demonstrated that Ca^2+^ mobilizing agents, such as ionomycin and glutamate, stimulate endogenous HO-2 activity in primary cortical culture. Results using a calmodulin mutant Phe 66 (a key amino acid in the calmodulin binding motif) showed that this Ca^2+^/calmodulin activation depends on the interaction between calmodulin and HO-2 and that this phenomenon is independent of CK2 activation. Apparently conformational changes in HO-2 induced by calmodulin binding are responsible for HO-2 activation. A similar conformational change in HO-2 could be occurring by phosphorylation of Ser 79 even both HO-2 activation mechanisms are independent and not additive [98]. The HO-2 activation by Ca^2+^/calmodulin-dependent mechanisms by glutamate has been also observed in piglet astrocytes [99]. Although actually it is not completely clear, it has been suggested that tyrosine phosphorylation stimulates HO-2 catalytic activity. Results showed that a PTK inhibitor reduces the HO-2 catalytic activity in cerebral microvessels, while the inhibition of protein tyrosine phosphatases increases it [100] (Figure 5(a)).

HO-2 activity can also be regulated by the presence of NO and ROS. Experiments with recombinant wt HO-2 and Cys 264/Cys 281→Ala/Ala mutant showed the inhibition of wt protein but not the mutant, suggesting NO binding to HRMs as responsible of HO-2 activity inhibition [101]. Later, it was described that glutamate increases NOS activity with the concomitant increase in NO, which in turn stimulates HO-2 activity. The hypothesis proposed is that this stimulation occurs through an increase in cGMP levels [102]. The same group has proposed that ROS work as regulators of HO-2 activity. In isolated cerebral vessels and cerebral vascular endothelial cells challenged with TNF-*α*, NADPH oxidase 4 (Nox4)-derived ROS increase HO-2 activity and CO production promoting survival of brain endothelial cells. These evidences support the hypothesis that HO-2 is a redox-sensitive enzyme posttranslationally activated by ROS through the thiol/disulfide redox molecular switch [103].

A specific potent activator of HO-2 is menadione (Vitamin K3). It increases HO-2 enzymatic activity up to 30-fold in an* in vitro* model. An increase in *V*_max⁡_ without changes in the apparent *K*_*m*_ was observed. Up to date, it is the only report that had identified a molecule that affects HO-2 activity specifically. Since menadione has no effect on the activity of HO-1 it has been suggested that it or its pharmacological offspring should be useful in the elucidation of physiological and pathological roles of HO-2 in the central nervous system (CNS) and other organs [104].

Regulation of catalytic activity of HO system is critical for survival processes in the cell. Two peculiar facts show that HO activity has many interesting unresolved issues. First, results with the inactive mutant HO-2-H45A and second, the HO1 : HO2 complex formation, with implications in HO activity. In the first case, Kim & Doré (2005) demonstrated that the addition of hemin to cells overexpressing wt or H45A mutant provokes increased cellular damage in H45A-cells due to its impossibility to metabolize heme. However, the use of H_2_O_2_ showed that HO-2H45A was also able to protect cells against oxidative stress injury, suggesting the multiplicity of action of the HO-2, besides its essential catalytic activity [105]. The other concern involves a physical interaction between HO-1 and HO-2. Using cell free systems and tissues, it was demonstrated that first 44 residues in HO-2 binds to 58–80 residues in HO-1 forming a HO-1 : HO-2 complex. This complex arrangement results in decreased activity of the HO system. Apparently, this interaction serves to limit HO activity in certain tissues such as brain. The regulation of HO enzyme activity by HO-1 binding to HO-2 could represent a useful negative feedback mechanism to limit HO activity when there is an excess of HO protein [106].

The main difficulty on HO activity regulation research and the specific contribution of HO-1 or HO-2, is the absence of specific inhibitors. In fact, the activity reported in many* in vivo *works is the result of total HO system. In the majority of cases, HO-1 or HO-2 activity is attributed to the abundance of each isoform in a particular tissue, or to the induction of HO-1 enzyme. Traditionally, competitive HO inhibitors such as the metalloporphyrins have been used owing to their structural similarity with heme [107]. However, the use of these inhibitors has been criticized since they are not selective between HO isoforms, leading some investigators to doubt the validity of the interpretation of the results obtained using these molecules. Despite the homology and high similarity between HO-1 and HO-2, proximal and distal helices in the apo- and heme-bound crystallographic data show that these particular differences open the possibility to design specific inhibitors. Recent reports have shown that imidazole-dioxolane compounds and other nonporphyrin molecules are selective inhibitors of HO isoenzymes; however a more specific inhibition has been observed for HO-1 isoform, promising powerful pharmacological tools to elucidate the regulatory characteristics of the HO system [108–110].

## 4. The Protective Role of HO-2

Cerebral tissue has a differential distribution of both isoenzymes. Although HO-1 is expressed barely in brain it has been detected in specific regions such as dentate gyrus, hipothalamus ventromedial, and any nucleus of brain stem. In contrast, HO-2 is expressed in abundant form in brain, where it is localized in neuronal populations in forebrain, hippocampus, midbrain, basal ganglia, thalamic regions, cerebellum, and brain stem. At cellular level, both isoenzymes have a different expression pattern due to the fact that HO-2 is expressed basically in neuronal populations, while HO-1 expression is markedly increased in glial cells and astrocytes by oxidative stress [111, 112]. It suggests that the two isozymes have individual roles, particularly in brain. HO-1 induction has been shown to selectively protect cultured cortical astrocytes, but not neurons, from oxidative stress resulting from exposure to hemoglobin and hydrogen peroxide [113, 114]. HO-2, on the other hand, is constitutively expressed in neurons throughout the brain and its expression has been shown to protect against apoptotic cell death in cortical, hippocampal, and cerebellar granule cultures and* in vivo* models of ischemic injury [16–18]. Besides, it has been suggested that HO-2 is responsible for the production of CO for physiological functions in neuronal populations and that is the first isoenzyme that responses to cellular damage in cerebral tissue due to its high expression in this organ.

A growing number of reports suggest the significant role of HO-2 in several* in vivo* and* in vitro* models [115]. First observations were reported by the group of Sylvain Doré who observed that neuronal damage or brain volume swell are increased in HO-2 (−/−) mice subjected to ischemia (in contrast to HO-1 (−/−) mice that did not show neuronal damage) [17] or in a model of intracerebral hemorrhage [116]. Intracranial injections of NMDA also showed increased neuronal damage in HO-2 (−/−) mice [17].

A traumatic brain injury model using HO-2 (−/−) animals shows HO-1 induction as a result of the imposed damage. However, a reduction in the total activity of HO system accompanied by cell loss in cortex, CA3 hippocampus region and lateral dorsal thalamus was observed, suggesting that HO-2 activity is crucial to protect neurons through heme catabolism [117].* In vivo *or* in vitro* deletion of HO-2 exacerbates inflammation induced damage [118, 119] and inflammatory molecules such as IL-1, IL-6 and NF-*κ*B are increased in aortic endothelial cells obtained from HO-2 null mice [120].

Basal levels of HO-2 participate not only in maintaining heme homeostasis but also in the cellular defense mechanisms against oxidative stress by regulating extracellular superoxide dismutase (EC-SOD), Akt, and apoptotic signaling kinase-1 (ASK-1), presumably by influencing the rate of heme degradation to BR, CO, and Fe^2+^. After silencing HO-2, a decrease in EC-SOD and phosphorylated Akt (Ser 473 and Thr 308) was observed affecting Bad and Bax regulation and increasing the proapoptotic protein ASK-1 in renal tissue of mice, suggesting that the control of EC-SOD, Akt, 3-NT, and ASK-1 by HO-2 is critical in the regulation of apoptosis [121].

Recently, it has been described that NO-induced apoptosis depends on the capability of cells to express HO system and p53. In vascular smooth muscle cells, NO significantly elevated HO-2 protein levels in wild-type p53 cells but not in knock-out p53 cells, without altering HO-2 mRNA levels in both cell types. Probably this change of HO-2 protein expression is due to translational or posttranslational regulation of HO-2 expression in a p53-dependent manner. Catalytic activity of HO is critically involved in the protective effect of p53 on NO-induced cell death, indicating that HO-mediated heme metabolites are important for cell protection [55] (Figure 1).

Other evidences that support a protective role of HO-2 in different models are the coordinated expression of BVR and HO-2 which promotes cardiomyocyte survival in response to isoproterenol [122]; the HO-2 involvement in the signal transduction pathway responsible to morphine tolerance through CO production [123]; and the coordinated expression and protein interaction of 6-phosphofructo-2-kinase/fructose-2,6-bisphosphatase 4 (PFKFB4) and HO-2 in glucose homeostasis in HepG2 human hepatome cells [124].

## 5. HO-2 and Hypoxia

### 5.1. HO-2 and Its Role in Oxygen Response

A unique function described for HO-2 is its role in oxygen sensing and hypoxic response in mammalian cells. In 1997, Maines proposed for the first time a heme/oxygen sensor function for HO-2 [8]. This proposal was based in the identification of the HRMs in HO-2 protein sequence [89] (see Section 3.3.1) and of the oxygen-sensing consensus sequence 5′TTTTGCA3′ in the 3′UTR of HO-2 mRNA. This sequence is located between the two poly A signals of the transcripts of HO-2 and is 100% identical to the oxygen-sensing consensus sequence of the erythrophoietin gen, which responds to variations in oxygen concentration [125]. This 3′ flanking region of the* Hmox2* contains many inverted repeats which enable the formation of stem-loops, that may participate in transcriptional regulation expression and at RNA level could bind proteins that stabilize or destabilize the mRNA. Shibahara et al. (2007) support the proposal of HO-2 as an O_2_ sensor based on morphometric changes observed for HO-2 −/− mice (reviewed in [24]).

Additionally, Williams et al. (2004) [70] and Yi and Ragsdale (2007) [69] have suggested that this oxygen sensor activity of HO-2 is related to the regulation of the activity of the carotid body BK_Ca_ channel, and that those actions are linked to redox signals as well as to heme and CO metabolism.

Carotid bodies are arterial chemoreceptors that sense changes in blood gases and respond to hypoxia events secreting acetylcholine, dopamine, and ATP [126–130]. These signals promote and increase rate and depth of ventilation as a response to systemic hypoxia [131]. The hypoxia response element within the carotid body is the glomus cell. K^+^ channels on the plasmatic membrane of the glomus cells are inhibited during hypoxia, promoting cell depolarisation, Ca^2+^ influx, and transmitter release. In rat glomus cells, two K^+^ channels have been implicated in the hypoxia-dependent depolarization, the tandem P-domain K^+^ channels family (TASK sub-type) [132], and the Ca^2+^-activated large conductance K^+^ channels BK_Ca_ [133].

BK_Ca_ channels are conductance Ca^2+^-activated K^+^ channels involved in neural firing, muscle contraction, hearing, and vascular tone modulation activated by membrane depolarization and the increase in intracellular Ca^2+^. Activation of BK_Ca_ channels repolarizes the cell membrane and leads to closing of voltage gated Ca^2+^ channels, working as a key negative feedback regulator of both membrane potential and intracellular Ca^2+^ levels (reviewed in [134]). BK_Ca_ channels are composed of four *α* subunits forming a pore, each subunit with two domains at the C-terminal named RCK1 and RCK2 that work as regulators of conductance for K^+^. RCK1 also serves as H^+^ sensor and appears to be involved in inhibition of the BK_Ca_ channel by heme and activation by CO [135, 136]. It has been proposed that heme, CO, and HO-2 bind to a linker region between the two RCK domains, named the heme binding domain (HBD) [137].

Based on observations that demonstrated that CO is capable of activating BK_Ca_ in carotid body [138], and the presence of HO-2 in the carotid body [139], Williams et al. (2004) demonstrated that HO-2 and BK_Ca_ channels have a physical interaction. Using electrophysiological assays with HEK293 cells, the authors demonstrated that CO modifies BK_Ca_ channels activity, supporting the notion that HO-2 activity is crucially involved in BK_Ca_ channel activity and regulation. The group of Kemp proposed a model in which O_2_ sensing is conferred upon the BK_Ca_ channel by colocalization with HO-2 [70]. In normoxia, HO-2 activity generates CO which is a channel activator and ensures that the BK_Ca_ channel is open at normal systemic O_2_ levels. Cellular CO levels are reduced during a hypoxic challenge as HO-2 substrate (O_2_) becomes scarce and CO production is dramatically reduced. This reduction in CO, in possible combination with direct heme-dependent inhibition evokes channel closure [70, 140].

More recently, it has been proposed that HRMs in HO-2 serve as a thiol/disulphide redox switch regulating binding of heme and that this switch in HO-2 responds to cellular oxidative stress and reductive conditions. Ragsdale group proposes that HRMs act as a “molecular rheostat” that responds to intracellular redox potential, controlling HO-2 activity by regulating heme binding in functions of hypoxic response in the carotid body [69, 94]. Results indicate that BK_Ca_ channels are regulated by a thiol/disulfide mediated redox switch as follows: (1) In normoxic conditions, O_2_ switches the thiol/disulfide of HO-2 and that of the BK_Ca_ channel to the disulfide state. In this state, HO-2 has high affinity for heme, in contrast to the low heme affinity of BK_Ca_ channel. It results in the opening of the channel, since heme is dissociated from the HBD or the CO generated by HO-2 is bound. (2) In hypoxic conditions, HO-2 loses affinity for heme, resulting in low heme degradation and CO production rates. The increase in local heme levels and decrease in CO concentrations allow the closing of BK_Ca_ channel (Figure 5(b)). In other words, authors propose that HO-2 functions as a sensor of acute reduction in environmental O_2_ by regulating BK_Ca_ channel activity changing the balance between intracellular heme concentrations, the production of CO, and cellular redox state [68, 69, 94].

### 5.2. HO-2 Response in Hypoxic Events

HO-2 deficient mice show hypoxemia, a blunted hypoxic ventilator response, hypertrophy of the pulmonary venous myocardium and enlargement of the carotid body, supporting the proposal that HO-2 participates in the oxygen response [24]. In the last years, several evidences suggest that HO-2 participates in the hypoxic response of certain tissues and cell types and that its expression is differentially regulated by oxygen tension.

HO-2 expression has shown to respond to hypoxia. For example, normobaric hypoxia decreased HO-2 protein by 40% in mouse liver after 7 days [141] and reduced levels in placenta of women that live in high altitude and chronic hypoxia have been reported [142]. Reduction of HO-2 as a consequence of hypoxic conditions has also been observed* in vitro*. Results obtained using different human cell lines show that HO-2 mRNA and protein are reduced [19]. They also demonstrated the half-life time of HO-2 transcript is reduced from 12 to 6 h without any effect on HO-1 mRNA. Since heme content was increased in some cell lines after 48 h hypoxia, they proposed that the observed reduction in HO-2 is an important adaptation of some human cell lines to maintain intracellular heme levels in oxygen reduction conditions. In addition, determination of HO-1 and HO-2 expression in mice subjected to normobaric hypoxia showed that there were no significant changes in the overall expression of HO-1 and HO-2 mRNAs and proteins in the lung during hypoxia. Nevertheless, immunohistochemical analysis showed that both isoforms were increased after 28 days of normobaric hypoxia in the pulmonary venous myocardium [141], suggesting a tissue specific differential regulation of HO system.

An interesting regulation of HO-2 expression has been described using human umbilical vein endothelial cells and human aortic endothelial cells subjected to hypoxia. He et al. (2010) observed that, under hypoxic conditions, HO-2 protein levels were maintained even 57% reduction in steady-state HO-2 mRNA level and 43% reduction in total protein synthesis were observed. Polysome profiling results revealed an increase of association of HO-2 transcript with polysomes during hypoxia. This association correlates with enhanced translation of HO-2 transcripts as a mechanism by which HO-2 protein levels are preserved. In fact, they observed that HO-2 maintains endothelial viability during hypoxic episodes, since inhibition of HO-2 expression provokes mitochondrial membrane depolarization, caspases activation, and apoptotic cell death [20].

In aortic endothelium of rats submitted to hypoxia, HO-2 protein increases, suggesting that endothelial modulation of rat aortic contraction to phenylephrine is mediated by HO-2. Apparently, this modulation is due to inhibition of ET-1 potentiation of *α*-adrenoceptor-induced contraction, suggesting the existence of an unrecognized mechanism by which HO inhibits systemic vascular reactivity in diseases that involve the absence or decrease of oxygen concentrations [143].

A significant increase in HO-2 protein expression was observed in hypoxia-induced animals, apparently due to the presence of a hypoxic global damage, which can upregulate vascular endothelial growth factor and promote the subsequent proliferation of endothelial cells. This result indicates that the observed HO-2 upregulation and the increase in HO-1 immunoreactivity is involved in the reduction of cell death induced by the absence of oxygen [144].

Finally, using electrophysiologic recordings a relationship between membrane excitability and HO-2 expression and activity in hypoxic conditions was observed. HO-2 is expressed in the hypoxia-chemosensitive regions of the rostral ventrolateral medulla (RVLM). Cardiorespiratory regions of the RVLM are excited by local hypoxia and RVLM neurons have shown to respond to NaCN and low O_2_ with either depolarization or hyperpolarization. The addition of HO inhibitor SnPP-IX blocks the depolarization response of hypoxia-excited neurons to both NaCN and low O_2_, but has no effect on the hyperpolarization response of hypoxia-depressed neurons. HO-2 is observed only in the hypoxia-excited neurons. These results suggest that RVLM neurons are excited by hypoxia via a HO-2-dependent mechanism. Apparently, hypoxic-induced increase in intracellular Ca^2+^ could enhance HO-2 activity by activating protein kinases (PKC/CK2). The products of HO reaction could modulate the conductance of ion channels and change neuronal excitability [145].

## 6. Concluding Remarks

In this review, we have addressed the issue of the HO system as a potent endogenous antioxidant cell defense system. The fact that HO-1 actively responds to stress conditions has meant this isoform as a protein of great interest to many groups. In contrast, since its discovery and characterization [58, 59], HO-2 has shown unique features. Despite the two isoforms homology, their almost identical primary and tertiary structures and the fact that both perform the same catalytic reaction, these isoforms present relevant differences at regulation of transcription, translation, and catalytic activity processes, as well as in their expression pattern at different cell types and tissues. These differences prompt intense investigation on their regulatory capacities at comparative levels.

Herein, we have also provided reviewed evidence that HO-2 participates in a multitude of housekeeping functions, mainly in brain, since it is the most prominent expressed isoform and the first to respond against oxidative stress. Indeed, the relevance of HO-2 for the CNS is emphasized by evidence showing that the continuous and regulated endogenous CO production by the activity of this enzyme is a key factor for maintaining the physiological function in neuronal cells and the vascular tone regulations of the cerebral blood vessels. Further support to this concept came from experiments with HO-2 deficient animals, demonstrating its involvement in brain cell damage produced by cerebral ischemia and intracerebral hemorrhage [16, 17]. For sure, enlightening studies will provide stronger evidence of this topic in a near future. In the meantime, some limitations should be considered in this process: as we have mentioned in the HO-2 activity section, the absence of specific pharmacological inhibitors for each isoform has complicated the inference of the independent contribution of each isoform to the studied models. However, important advances have been obtained from imidazole-dioxolane derivatives [110–112]. Therefore, more attention shall be paid to the production of new and more specific inhibitors of both HO isoforms if we want to bring an accurate pharmacological approach to the role of these enzymes in the physiology of the CNS. As a temporary shortcut, in recent years, the use of siRNAs has shown to importantly contribute to clarify the role of each isoform in different models.

With respect to the described novel function of HO-2 as O_2_ sensor, this issue opens a refreshing line of research to be explored in the next years. In this regard, future research shall clarify in a more precise manner all those functions that HO-2 already exerts. Moreover, it is pertinent to point out that even when HO-2 does not respond to the numerous stimuli compared with HO-1—mainly due to the absence of regulatory elements in its promoter—its expression at mRNA and protein levels is influenced by hypoxia conditions. This evidence might suggest that the role of HO-2 as a constitutive enzyme is questionable, especially during the occurrence of events related with varying oxygen levels, and this is particularly relevant for our knowledge of the pathological processes affecting the CNS, particularly those related with hypoxia, ischemia, hypertension, and instabilities of respiration. Thus, the role of HO-2 as a sensor of oxygen concentrations will shape future research on this field.
